# Electrochemical Impedance Analysis of Biofunctionalized Conducting Polymer-Modified Graphene-CNTs Nanocomposite for Protein Detection

**DOI:** 10.1007/s40820-016-0108-2

**Published:** 2016-09-15

**Authors:** Shobhita Singal, Avanish K. Srivastava, Rajesh

**Affiliations:** 1 CSIR-National Physical Laboratory, Dr. K. S. Krishnan Road, New Delhi, 110012 India; 2 Academy of Scientific and Innovative Research (AcSIR), CSIR-National Physical Laboratory, Dr. K. S. Krishnan Road, New Delhi, 110012 India

**Keywords:** Conducting polymer, Graphene, Carbon nanotube, Hybrid, Transducer, Protein antigen cTnI, Electrochemical impedance

## Abstract

We report an electrodeposited poly(pyrrole-co-pyrrolepropylic acid) copolymer modified electroactive graphene-carbon nanotubes composite deposited on a glassy carbon electrode to detect the protein antigen (cTnI). The copolymer provides pendant carboxyl groups for the site-specific covalent immobilization of protein antibody, anti-troponin I. The hybrid nanocomposite was used as a transducer for biointerfacial impedance sensing for cTnI detection. The results show that the hybrid exhibits a pseudo capacitive behaviour with a maximum phase angle of 49° near 1 Hz, which is due to the inhomogeneous and porous structure of the hybrid composition. The constant phase element of copolymer is 0.61 (*n* = 0.61), whereas, it is 0.88 (*n* = 0.88) for the hybrid composites, indicating a comparatively homogeneous microstructure after biomolecular functionalization. The transducer shows a linear change in charge transfer characteristic (*R*_et_) on cTnI immunoreaction for spiked human serum in the concentration range of 1.0 pg mL^−1^–10.0 ng mL^−1^. The sensitivity of the transducer is 167.8 ± 14.2 Ω cm^2^ per decade, and it also exhibits high specificity and good reproducibility.

**Electronic supplementary material:**

The online version of this article (doi:10.1007/s40820-016-0108-2) contains supplementary material, which is available to authorized users.

## Introduction

In recent years, enormous interest has been attached to hybrid nanocomposites due to their wide applications in environmental science, energy conversion, sensing, etc. [1]. Recently, theoretical and experimental reports on hybrid carbon nanostructures have brought special interest in design and fabrication of conducting polymer/carbon nanostructured composites [2, 3]. The interest is motivated by their extraordinary physical properties and remarkable electrocatalytic properties. Polypyrrole (PPy), a conducting polymer with high conductivity and excellent biocompatibility, has been widely used as a functional material in the development of electrochemical sensors [4–8]. However, it has poor permeability and hydrophobicity that obstructs the diffusion of analyte, leading to a fall off sensitivity of the immunosensor. Another major drawback is associated with its instability due to swelling and exfoliation with times upon electrochemical modulation and/or solution soaking [9]. However, the deposition of PPy film on highly conductive and rigid material like graphene or metal nanoparticle is proved to be an efficient way to enhance the electrochemical/mechanical stability of the polymer film without compromising its electrochemical activity [10, 11].

Carbon nanomaterials, including carbon nanotubes (CNTs) and graphene, have shown great potential in biosensing due to its excellent electric/thermo conductivity, good mechanical/chemical stability, high surface area, and unique physical/chemical properties [12–17]. In graphene, these excellent properties only emerge in the 2D planar direction, thereby limiting its scope and applications. New efforts in graphene researches have attempted to address this weakness, wherein graphene acts as a platform for anchoring other nanomaterials. CNTs, whose properties emerge in the axial direction, can be functionalized onto the surface of graphene, which can combine unique properties of two carbon allotropes in all directions and increase active surface areas and faster electron transfer. Recent studies predict that three dimensional graphene-CNTs (G-CNTs) nanostructure possesses desirable out-of-plane properties when maintaining in-plane properties. It is attractive for numerous innovative applications, including as an efficient transducer for sensor applications [18–22].

Cardiac troponin I (cTnI), a protein subunit of cardiac troponin complex is extensively used as diagnostic marker of acute myocardial infarction (AMI) [23]. Owing to its small size (29 kDa), cTnI rises rapidly in the blood stream within 3–4 h after onset of AMI and remains to be raised up to 10–14 days. This will result in a long diagnostic window for the detection of AMI. Compared with other biomarkers of myocardial injury, such as myoglobin, C-reactive protein and creatine kinase-MB isoenzyme, cTnI has excellent cardio specificity and selectivity for cardiac damage other than damage to skeletal muscle or other organs. A cTnI level of 0.1–2.0 ng mL^−1^ indicates to an unstable angina and other heart disorder, whereas a level greater than 2.0 ng mL^−1^ places a patient in high-risk category for early adverse outcomes [24, 25]. This underlines the need to develop a sensitive diagnostic tool for quantitative detection of cTnI for diagnosis of AMI.

In the present work, we demonstrate the electrochemical synthesis of conducting copolymer PPy-PPa over G-MWCNT hybrid film deposited on a glassy carbon electrode (GCE), as an impedance immunosensor, for ultrasensitive detection of cardiac troponin I (cTnI) spiked in human serum. The surface morphology and electrochemical properties of the hybrid were characterized. The analytical performance of the bioelectrode was investigated for the quantitative detection of cTnI in spiked human serum using charge transfer characteristic (*R*_et_) as a sensing element in electrochemical impedance spectroscopy. The utilization of the changes observed in the *R*_et_ provided a good correlation with antigen concentration in the low-frequency region (<1 Hz), which helps in preserving the micro environment for biomolecular reactions useful for both in-field and at-line applications.

## Experimental

### Chemicals and Reagents

Human cardiac troponin I (cTnI: Cat 8T53), human anti-cardiac troponin I (anti-cTnI: Cat 4T21 MAb19C7), C-reactive protein (CRP: Cat 8C72), and myoglobin (cMb: Cat 8M50) were obtained from Hytest (Turku, Finland). *N*-(3-dimethylaminopropyl)-*N*′-ethyl carbodiimide hydrochloride (EDC), *N*-hydroxysuccinimide 98 % (NHS), pyrrole, pyrrolepropylic acid, and *p*-toulenesulfonic acid (*p*TSA) were obtained from Sigma-Aldrich Corp. All other chemicals were of analytical grade and used without further purification.

### Instruments

The surface morphologies and electrochemical characterizations of the bioelectrode were carried out using scanning electron microscopy (SEM, SUPRA40 VP, Germany), high-resolution transmission electron microscopy (HRTEM, Technai G2F30 STwin, The Netherlands), Raman spectroscopy (Renishaw Raman spectrometer, Germany), and energy dispersive X-ray analysis (EDAX, SUPRA40 VP, Germany). Electrochemical impedance spectroscopic (EIS) measurements were done on an AUTOLAB instrument from Eco Chemie (PGSTAT302N, The Netherlands). The EIS measurements were conducted in 0.1 M phosphate buffer solution (PBS) (pH 7.4, 0.1 M KCl) containing a mixture of 2.0 mM K_3_[Fe(CN)_6_] and 2.0 mM K_4_[Fe(CN)_6_]. The EIS parameters were obtained by circuit fitting the EIS experimental data using GPES (General purpose electrochemical system version 4.9, Eco Chemie) software. All electrochemical measurements were carried out in a conventional three-electrode cell configuration consisting of a PPy-PPa/G-CNTs/GCE as a working electrode, Ag/AgCl as a reference electrode, and platinum wire as a counter electrode.

### Preparation of the Bioelectrode

The G-CNTs hybrid films were synthesized by the chemical vapour deposition (CVD) technique as reported earlier [26]. In brief, the G-CNTs hybrid was grown on a copper foil deposited ~1 nm thickness of Fe film using e-beam evaporator. The Fe-deposited copper foil was placed in the fused silica tube (5 cm in diameter and 100 cm in length) and the temperature was raised to 750 °C under 200/100 sccm Ar/H_2_ atmosphere flow. A 10 sccm flow of acetylene (used as a gaseous carbon source) was introduced for 20 min once the temperature stabilized at 750 °C, followed by cooling of the furnace to the room temperature in Ar/H_2_ atmosphere flow. The growth time of 20 min is the most optimized at which the CNTs are uniform all over the graphene surface. If the time is less than 20 min (e.g., 10 or 15 min), the CNTs are un-uniformed. Whereas, if the time is more than 20 min, it will get a high density CNTs, which could not hold firmly onto the GCE surface and peel off during experimental measurements.

**Fig. 1:**
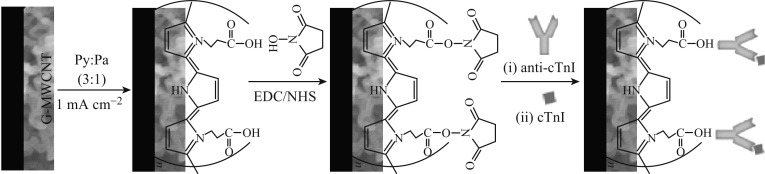
Fabrication scheme of anti-cTnI-PPy-PPa/G-CNTs/GCE bioelectrode

For the preparation of PPy-PPa/G-CNTs nanocomposite, GCE (3 mm in diameter) was first polished with respective 1, 0.3, and 0.05 µm alumina slurry using a polishing cloth to produce a mirror-like surface, and then it was rinsed ultrasonically with double distilled water and ethanol for 3 min each, and dried with a high purity N_2_ gas flow. G-CNTs hybrid film was transferred over GCE by scooping, followed by drying in oven at 50 °C for 1 h. The G-CNTs-modified GCE were anodized at 1.7 V for 500 s in PBS (pH 7.4, 0.1 M KCl), followed by cathodization at −0.6 V for 60 s to obtain an electroactive G-CNTs. The PPy-PPa copolymer was electrochemically deposited on the G-CNTs/GCEs in a degassed (N_2_ purged) aqueous solution containing 0.1 M pyrrole, 0.03 M pyrrolepropylic acid, and 0.1 M *p*TSA at a fixed current density of 1 mA cm^−2^ by inducing a total charge density of 100 mC cm^−2^. The most optimum-induced charge density of 100 mC cm^−2^ was chosen based on the facts that at low density (<100 mC cm^−2^), the polymer film is unstable, whereas, at large density (>100 mC cm^−2^), the electroactive polymer surface is too small. Finally, the PPy-PPa/G-CNTs/GCE was biofunctionalized with anti-cTnI by incubating with an aqueous solution containing 0.03 M NHS and 0.15 M EDC for 1 h and then with 10 µL PBS containing 100 µg mL^−1^ anti-cTnI at 4 °C for 2 h, followed by washing with PBS and drying under N_2_ gas flow. The biofunctionalized electrode was then incubated in 0.1 % bovine serum albumin (BSA) solution (w/v) to block the non-specific binding sites, if any, on the electrode surface, followed by washing with PBS, to remove the physically adsorbed antibody and drying under N_2_ gas flow to obtain the desired anti-cTnI-PPy-PPa/G-CNTs/GCE bioelectrode. The as-prepared bioelectrode was stored at 4 °C when not in use. The stepwise fabrication of the bioelectrode is schematically represented in Fig. 1.

## Results and Discussion

### Microstructural Characterization of PPy-PPa/G-CNTs Nanocomposite

**Fig. 2:**
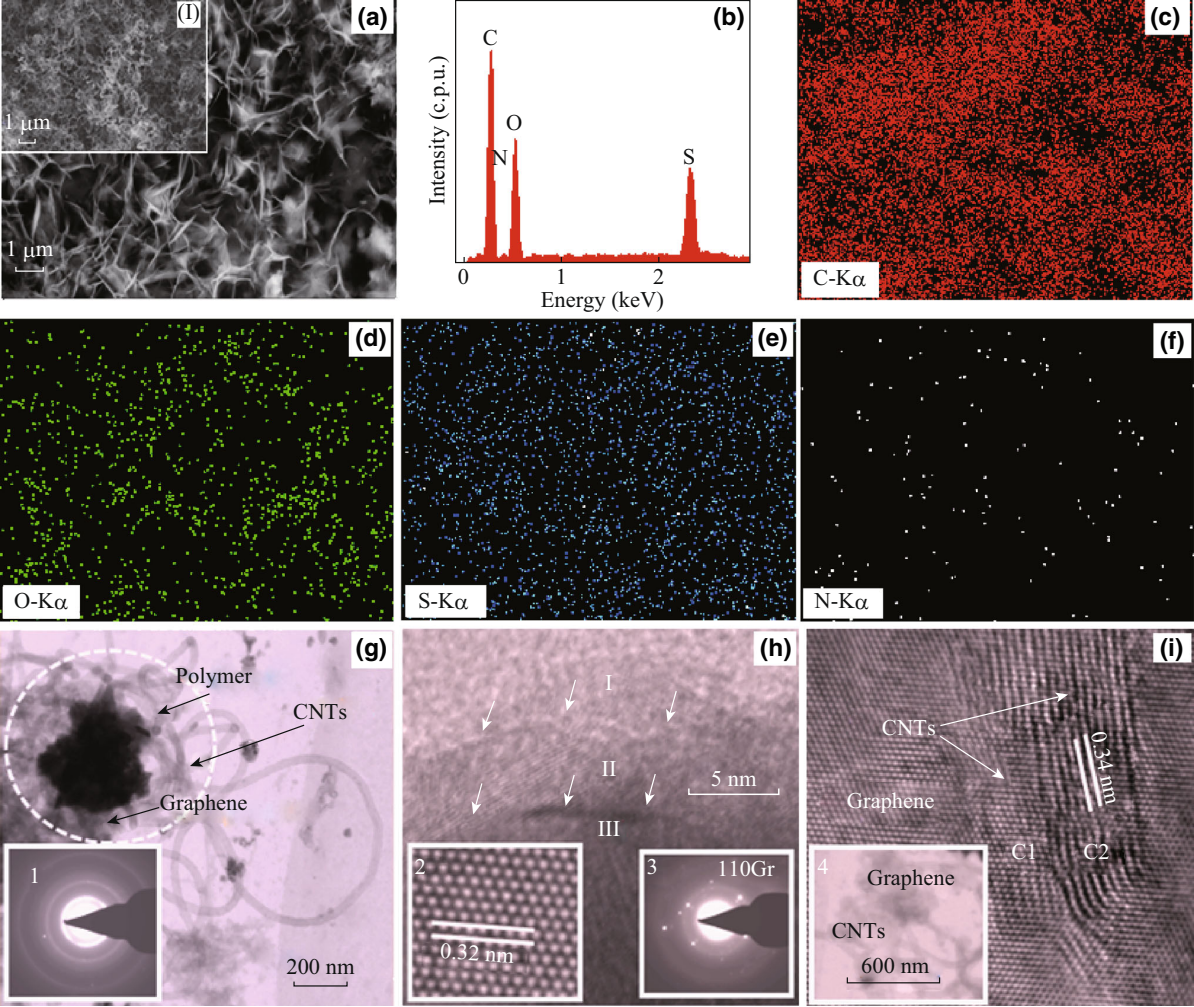
**a** SEM image, **b** EDAX spectrum, **c**–**f** elemental map, **g**–**i** HRTEM images of PPy-PPa/G-CNTs composite. *Insets* (I) SEM image of G-CNTs, (1) SAED pattern of the composite, (2) Atomic scale image of graphene, and (3) SAED pattern of graphene, (4) TEM image of interface between graphene and CNTs at initial growth stage

Figure 2a shows the SEM image of G-CNTs after the PPy-PPa copolymer electrodeposition (the inset is G-CNTs image). It can be seen in the inset that CNTs are highly dense and uniformly distributed on the graphene surface. After copolymer electrodeposition, three-dimensional petal-like structure was formed uniformly across the G-CNTs hybrid surface. This was further confirmed by EDAX spectra, showing the characteristic peaks (Fig. 2b), and elemental mapping (Fig. 2c–f) corresponding to energy levels of carbon (0.277 keV), nitrogen (0.392 keV), oxygen (0.525 keV), and sulphur (2.307 keV). The presence of N, O, and S in the elemental analysis due to amine, carboxyl, and sulphonate groups in PPy-PPa copolymer, revealed the uniform electrodeposition of copolymer throughout the surface of G-CNTs.

HRTEM images of PPy-PPa/G-CNTs composite are displayed in Figs. 2g–i. The polymer, graphene, and CNTs were observed (Fig. 2g) clearly. In the encircled region of Fig. 2a, the gray-level contrast may be corresponding to graphene, whereas, the petal shape is from PPy-PPa which has a clear distinct gray-level difference from CNTs and graphene due to its thick polymer-chain composition. Selected area electron diffraction (SAED, inset 1 in Fig. 2g) pattern exhibits a set of diffused Debye rings taken from the encircled composite. Due to the complexity of the hybrid (graphene, CNTs and PPy-PPa) with several interfaces and grain boundaries, it is difficult to interpret the pattern further. However, it still can infer that the composite has a major fraction of crystallinity along with a minor amorphous structure. At high magnifications, the presence of graphene is clear and multilayered graphene is confirmed. In Fig. 2h, three layers of graphene (marked as I, II, III) are overlapped. At the atomic scale, a typical honeycomb structure with the fringe separation of about 0.32 nm is clearly observed (inset 2 in Fig. 2h). The SAED from graphene exhibits a hexagonal arrangement of diffraction spots in reciprocal space (inset 3 in Fig. 2h). Miller indices of (110) plane corresponding to a hexagonal crystal structure is marked on the SAED pattern. Figure 2i displays CNTs structure overlapped in two nanotubes, marked as C1, C2 on nano-sheets of graphene. The individual walls of the nanotubes of interlayer separation about 0.34 nm are stacked with a very coherent microstructure of graphene throughout the region. The TEM image of the interface between graphene and CNTs (inset 4 in Fig. 2i) shows the growing feature of the CNTs on the graphene floor during initial CVD growth process. Raman spectroscopy shown in Fig. S1 exhibits the characteristic 2D and G peaks with an intensity ratio (*I*_2D_/*I*_G_) of 0.35, and further revealed a trilayer graphene thickness in G-CNTs hybrid.

### Electrochemical Characterization of PPy-PPa/G-CNTs and the Bioelectrode

**Fig. 3:**
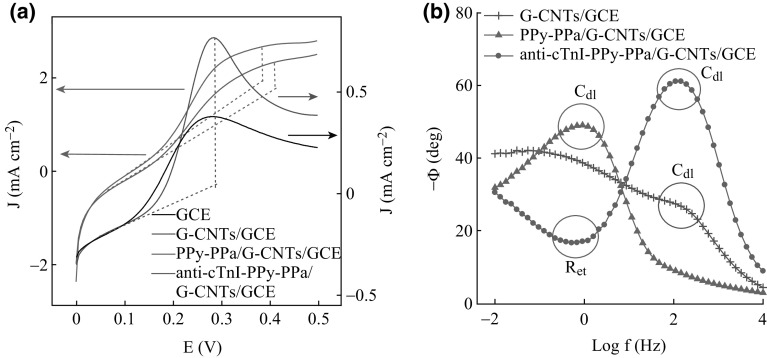
**a** Linear sweep voltammogram of the hybrid bioelectrode at different stages of modification surface. **b** Corresponding Bode plot

Linear sweep voltammetry (LSV) is highly efficient and sensitive technique to investigate the electron transfer properties of the electrode surface, and it was utilized to characterize the anti-cTnI-PPy-PPa/G-CNTs/GCE electrode here. The linear sweep voltammetric measurements were performed over the voltage range of 0–0.5 V versus Ag/AgCl as reference electrode in PBS containing 2.0 mM [Fe(CN)_6_]^3−/4−^ at a scan rate of 50 mV s^−1^. Figure 3a shows anodic peak in LSV conducted on the electrode at various stages of surface modifications. The current density (*J*) is 0.38 mA cm^−2^ for bare GCE and it increases to 0.72 mA cm^−2^ for the electroactive G-CNTs/GCE. This is due the increased electron transfer characteristics resulted from the edge plane defects at G-CNTs, which generated from the oxygenated species during electrode anodization. After electrodeposition of PPy-PPa copolymer on G-CNTs/GCE electrode, the value of *J* decreases to 0.65 mA cm^−2^ with a large background current [27]. This may be attributed to the presence of negatively charged –COOH group of PPa, which generates a repulsive force towards the negatively charged redox probe at the electrode/solution interface, and thus reduces the ion perturbation. The electroactive surface area of G-CNTs hybrid film was found to be 16.1 × 10^−5^ cm^2^ calculation using Randles–Sevcik equation, which slightly decreases to 14.5 × 10^−5^ cm^2^ after modification with the PPy-PPa. The detail is described in supporting information. After biomolecular immobilization with anti-cTnI and subsequent passivation of the electrode with a blocking reagent BSA, further decreases in *J* values were observed to be 0.54 and 0.42 mA cm^−2^, respectively. This may be explained on the basis of the insulating and highly organized hydrophobic layer of protein molecules at the bioelectrode surface, which could hinder the access of the redox probe on electrode surface and thus result in sluggish electron transfer kinetics.

The above configuration was further characterized by EIS. Briefly, in EIS theory, a Nyquist or Bode plot is mainly composed of three regions: the high-frequency part corresponding to the solution properties, the middle-frequency part corresponding to the electrode/solution interface (i.e., capacitive properties), and the low-frequency part corresponding to the charge transfer characteristics of the electrode surface. The complex impedance can be represented by Eq. 1,

1 $$Z^{\prime} = Ze^{i\varPhi } ,$$

where the magnitude *Z* represents the ratio of the voltage difference amplitude to the current amplitude and acts like resistance, and *Φ* gives the phase difference between the voltage and the current. The change in the phase shift (angle *Φ*) in the Bode plot was chosen as a prominent EIS element in frequency region of 0.01–10, 000 Hz as shown in Fig. 3b. The G-CNTs electrode shows two phase shifts of *Φ* = 26.6° and 39.2° at frequency of ~162 and ~1 Hz, respectively. These phase shifts at higher and lower frequency correspond to capacitive and diffusive characteristics of the G-CNTs hybrid. However, a single-phase shift (*Φ* = 49°) at frequency of ~1 Hz was observed for the PPy-PPa copolymer modified G-CNTs, which corresponds to only a capacitive behaviour with no diffusive characteristic, indicating a moderate heterogeneous feature of the modified electrode compared with its native G-CNTs. This was further enunciated by another impedance parameter of constant phase element *n*, which accounts for the surface inhomogeneity of the electrode surface. The value of *n* = 1 represents a smooth defect free surface, whereas 0 < *n* < 1 represents porous and rough surface [28]. The high value of *n* = 0.61 observed on PPy-PPa/G-CNTs compared with n = 0.44 on native G-CNTs further indicates a slight reduction in surface inhomogeneity in the polymer-modified electrode, which is later found to be nearly homogenous (*n* = 0.88) upon biomolecular immobilization with anti-cTnI. The same is reflected in the change of phase angle, where the maximum phase shift (*Φ* = 61°) obtained in G-CNTs at frequency of ~1.0 Hz is moved to a high region (>100 Hz). A lowest phase shift (where *v*/*t* and *I*/*t* are nearly in phase) with *Φ* = 29° appears at the same frequency, corresponding to a charge transfer characteristic of a homogeneous surface after biomolecular immobilization.

### EIS Response of the Bioelectrode to Protein Antigen cTnI

When biomolecular probe (antibody) forms a complex with the complementary antigen molecule upon immunoreaction on the bioelectrode surface, it will cause an increase in charge transfer resistance that leads to a decrease in the phase shift (lowest phase angle) of the circuit in low-frequency region [29]. The anti-cTnI-PPy-PPa/G-CNTs/GCE bioelectrode was incubated with a 10 µL sample of cTnI-spiked human serum for 10 min at room temperature, followed by washing with PBS and drying under N_2_ gas flow. Impedance analysis was carried out in PBS solution containing 2.0 mM [Fe(CN)_6_]^3−/4−^.

**Fig. 4:**
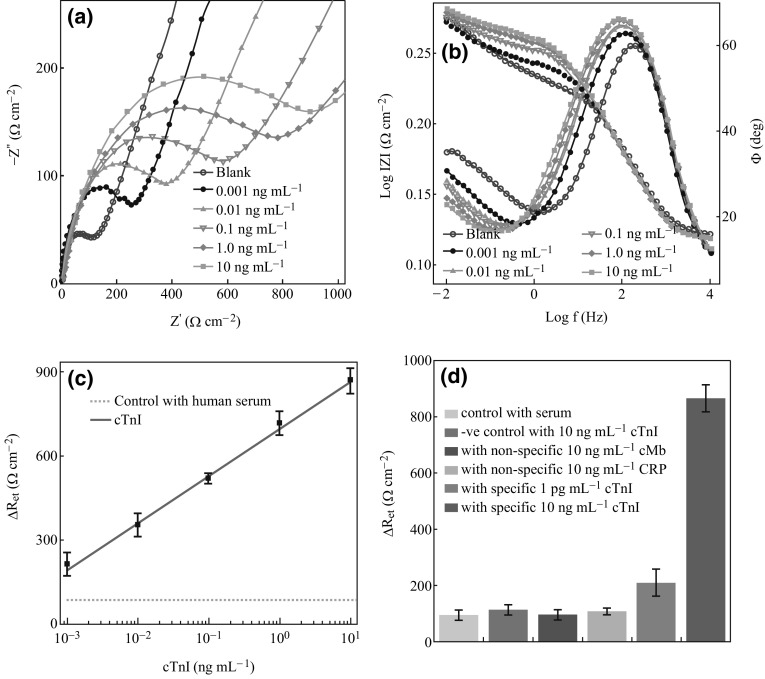
**a** Nyquist plot of the bioelectrode before and after incubation with different cTnI concentrations in human serum. **b** Corresponding Bode plot. **c** Concentration-dependent calibration curve of the bioelectrode. **d** Specificity of the bioelectrode towards cTnI with multiple controls

Figure 4 shows the Nyquist and the Bode plot of the bioelectrode with different cTnI concentrations. The diameter of the semicircle in the Nyquist plot of the bioelectrode (Fig. 4a) is gradually increased with increasing concentration of the dispensed cTnI, indicating an increasing antigen–antibody complex formation. This will result in a large electron transfer resistance (*R*_et_) at the electrode surface. Since the changes in *R*_et_ values were found to be much more prominent than those of other impedance components (Table 1), it was taken as a suitable sensing parameter. Though, not many changes were observed in the *Z*_w_ values, noticeable changes in *Y*_o_ in a decreasing order show decreasing capacitive behaviour on subsequent immunoreaction with increasing concentration of cTnI. This may be due to the increasing coverage of electrode surface with increasing cTnI concentration and decreasing its exposure to electrolyte [30]. The immunoreaction was further investigated by corresponding Bode plot (Fig. 4b). It is interesting to note that the lowest phase angles on immunoreaction were observed at low-frequency region of 0.1–1.0 Hz, which corresponds to an increasing *R*_et_ feature of the bioelectrode, indicating to a good probe (anti-cTnI) orientation and biocompatible feature of the electrode.

**Table 1:** EIS characteristic parameters of the bioelectrode on immunoreaction with different concentrations of target cTnI

cTnI concentration (ng mL^−1^)	*R*_et_ (Ω cm^2^)	CPE Y_o _(mF cm^−2^)	*n*	*Z*_w_ (×10^−5^) (*Ω* cm^2^)	*χ* ^2^ (×10^−2^)	RSD (%) in ∆*R*_et_ (*n* = 3)
Blank	109.2	1.98	0.824	5.25	3.62	–
0.001	298.6	1.61	0.843	4.78	3.59	13.4
0.010	416.5	1.42	0.844	4.70	5.37	11.3
0.10	616.0	1.35	0.793	4.76	6.53	2.8
1.00	793.1	1.26	0.791	4.73	5.36	6.0
10.0	925.4	1.13	0.797	4.73	4.84	5.3

Figure 4c shows the calibration curve of the bioelectrode. One can see a linear relationship between the changes in charge transfer resistance i.e., before (blank) and after immunoreactions [∆*R*_*et*_ = (*R*_*et*_)_after immunoreaction_ − (*R*_*et*_)_blank_], and the cTnI concentration is in the range of 0.001–10 ng mL^−1^, which can be represented by Eq. 2,

2 $$\Delta R_{\text{et}} \left[ {\text{cTnI}} \right] \, = \, b\left[ {\text{cTnI}} \right] \, + \, 695.8 \, \pm \, 18.96.$$

The bioelectrode shows *R*_et_ sensitivity (slope ‘b’ of the calibration curve) of 167.8 ± 14.2 Ω cm^2^ per decade of cTnI. The analytical performance of the bioelectrode is compared with the previous reports (Table 2) and was found to be better in terms of linear dynamic range and lowest detection limit [2, 31–36]. This better performance of the bioelectrode may be attributed to the high cTnI probe loading due to active pendant carboxyl binding sites of the conducting PPy-PPa and electroactive behaviour of the G-CNTs hybrid, resulting in enhanced electron transfer at the electrode solution interface of the bioelectrode on immunoreaction.

**Table 2:** Comparison of analytical performance of the bioelectrode with other existing biosensor

Sensing technique	Transducing matrix	Detection range	Detection limit	References
Amperometry	PDMS/Au	0.2 ng mL^−1^–10.0 µg mL^−1^	148.0 pg mL^−1^	[2]
Impedimetric	Carbon nanofiber	0.25–1.0 ng mL^−1^	0.2 ng mL^−1^	[31]
Surface plasmon resonance	Au nanorods	–	10.0 pg mL^−1^	[32]
Potentiometry	Au/ITO	1.0–100.0 ng mL^−1^	–	[33]
Stripping voltammetry	Ag/SPE	0.1–32.0 ng mL^−1^	0.10 ng mL^−1^	[34]
Colorimetric	PDMS/Au	0.01–10.0 ng mL^−1^	0.01 ng mL^−1^	[35]
Lateral flow assay	Magnetic beads	–	0.01 ng mL^−1^	[36]
Impedimetric	PPy-Ppa/G-CNTs	1.0 pg mL^−1^–10.0 ng mL^−1^	1.0 pg mL^−1^	Present work

The specificity of the bioelectrode towards specific cTnI was examined by monitoring the responses from multiple controls such as native normal human serum, non-specific biomarkers C-reactive protein (CRP), myoglobin (cMb) spiked in human serum, and a negative control with a native electrode (PPy-PPa/G-CNTs/GCE) without anti-cTnI immobilization. The comparative response of the bioelectrode with multiple controls with respect to the specific cTnI under identical condition is shown in Fig. 4d. The response of Δ*R*_et_ = 98.6 ± 13.0 and 111.3 ± 7.3Ω cm^2^ obtained for 10 ng mL^−1^ individuals of respective cMb and CRP-spiked serum are comparable to the pure normal human serum response (Δ*R*_et_ = 95.9 ± 17.2 Ω cm^2^), but they are far below the response (Δ*R*_et_ = 869.7 ± 46.4 Ω cm^2^) of bioelectrode to the same concentration of cTnI-spiked serum. This has further been confirmed with the negative control which also shows a comparable response (Δ*R*_et_ = 119 ± 11.6 Ω cm^2^) nearby to that of the pure human serum. The response of Δ*R*_et_ = 220 ± 29.7 Ω cm^2^ to the lowest 1 pg mL^−1^ cTnI concentration is about 2.3 times higher than the corresponding response to pure human serum. This lowest detection of cTnI is much smaller than the reported lowest detection level of 5.0 ng mL^−1^ cTnI from the conventional ELISA method [31], which is signified the importance of the conducting polymer as a biomolecular linker over a nanocarbon hybrid for a better probe orientation. These results predicted a good specificity of the bioelectrode for cTnI. The reproducibility of the bioelectrode was evaluated in terms of relative standard deviation measured at individual cTnI concentration on three different bioelectrodes prepared in the same manner under identical conditions. It is shown as error bars in the calibration curve.

The measured RSD values of ~2.8–13.4 % at individual cTnI concentration were found to be within the acceptable error range under the tested conditions, suggesting a reproducible assay. The stability of the bioelectrode in solution was also investigated by taking repeated measurements (5–6 times) with a fixed concentration (1.0 ng mL^−1^) of cTnI sample. It was found to be within the reasonable range of consistent response, indicating to a good bioelectrode stability in solution.

## Conclusion

We report a hybrid PPy-PPa/G-CNTs/GCE bioelectrode for ultrasensitive detection of cTnI. The pendant carboxyl groups of the polymer allowed efficient biomolecular immobilization of anti-cTnI on the electroactive G-CNTs, which is suitable for fabrication of a low-frequency impedimetric immunosensor. The modification of G-CNTs with conducting polymer makes prominent changes in the surface morphology and electrochemical characteristics with a capacitive behaviour without any diffusive feature. This biomolecular immobilization was modified to a nearly homogenous surface (*n* = 0.88) with a dominant charge transfer characteristic at the same frequency of 1.0 Hz. It is useful for the quantitative analysis of analyte at low-frequency region. The synergistic combination of physical and electrochemical characteristics of both the conducting polymer and 2D-graphene with pillared G-CNTs hybrid results into the detection of cTnI concentration as low as 1.0 pg mL^−1^ (34.4 fM) in spiked human serum, and the sensitivity is high of 167.8 ± 14.2 Ω cm^2^ per decade of cTnI. Our results may be a reliable foundation of impedance immunosensor in detection of enzyme, protein, or DNA.

## Electronic supplementary material

Below is the link to the electronic supplementary material.

Supplementary material 1 (PDF 94 kb)
