# Supplementary material for: Electrochemical Impedance Analysis of Biofunctionalized Conducting Polymer-Modified Graphene-CNTs Nanocomposite for Protein Detection
Source: Nanomicro Lett. 2016 Sep 15;9(1):7. doi: 10.1007/s40820-016-0108-2 (PMC6223772; doi:10.1007/s40820-016-0108-2)
Supplement: Supplementary file 1 — Supplementary material 1 (PDF 94 kb) [file 40820_2016_108_MOESM1_ESM.pdf]

Supporting Information for

Electrochemical Impedance Analysis of Biofunctionalized Conducting Polymer modified Graphene-CNTs Nanocomposite for Protein Detection

Shobhita Singal[1,2], Avanish K Srivastava[1], Rajesh[1,2,*]

[1]CSIR-National Physical Laboratory, Dr. K.S. Krishnan Road, New Delhi-110012, India

[2]Academy of Scientific and Innovative Research (AcSIR), CSIR-National Physical Laboratory, Dr. K.S. Krishnan Road, New Delhi-110012, India

*Corresponding author.E-mail: rajesh_csir@yahoo.com

Raman Spectra of G-MWCNT Hybrid

Figure S1 shows the acquired Raman spectra of the hybrid on copper foil. The D peak is observed at 1358 cm^{-1}, arising due to the presence of structural disorder such as grain boundaries, in the G-MWCNT hybrid. The second peak is the G peak, observed at 1588 cm^{-1}, corresponds to the C-C bond stretching vibration in the graphitic plane. The third common peak, commonly referred as 2D peak appears due to the second order vibrations of the C-C bond and provides information about the two dimensional graphitic stacking of the carbon material.

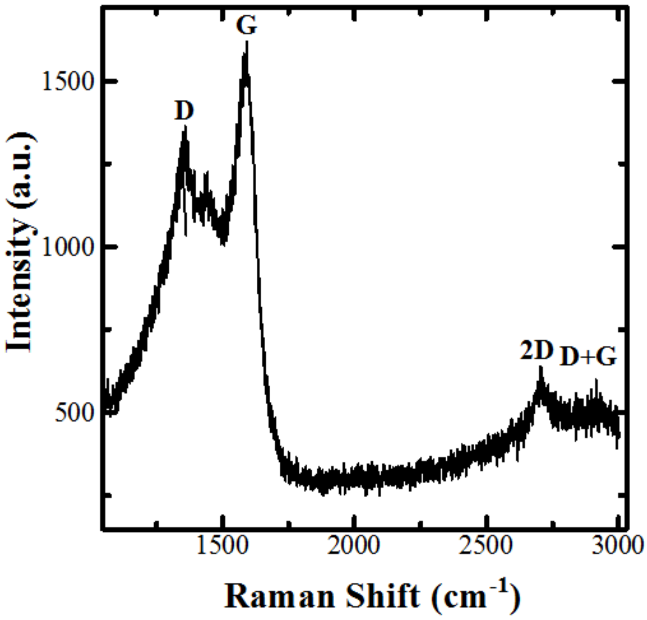

Fig. S1 Raman spectra of G-CNTs hybrid on a Cu foil

The electroactive surface area of G-CNTs hybrid film both before and after the electrodeposition of copolymer film was calculated using Randles-Sevcik equation:

$I_p = 2.69 \times 10^5\, AD^{1/2}n^{3/2}v^{1/2}C$

where $n = 1$, is the number of electrons participating in the redox reaction, A is the electroactive surface area (cm^2), $D = 6.70 \times 10^{-6}$ $cm^2\ s^{-1}$, is the diffusion coefficient of $[Fe(CN)_6]^{3-}$ in solution, $C = 0.002$ M corresponds to the concentration of the redox probe ($K_3[Fe(CN)_6]$), and v is the scan rate of the potential perturbation ($V\ s^{-1}$). The electroactive surface area for G-CNTs and polymer modified G-CNTs hybrid film were found to be 16.1×10^{-5} and 14.5×10^{-5} cm^2.
